# Application of Hybrid Multiple Attribute Decision-Making Model to Explore the Design Strategies of Children's Facilities in Neighborhood Open Spaces Based on Sensory Integration Theory

**DOI:** 10.1155/2021/5556172

**Published:** 2021-05-29

**Authors:** Ge Wang, Ruo-Ying Wang, Ting-Lan Liu, Ying Zhuo, Kang Shen

**Affiliations:** School of Architecture and Applied Art, Guangzhou Academy of Fine Arts, Guangzhou 511400, China

## Abstract

In recent years, the prevalence of sensory integration disorders in children in urban areas has increased. Most existing sensory integration treatments are located in hospital-based sensory integration units; however, medical resources are extremely limited, making it difficult to guarantee the appropriate treatment time and intervention results for many children. The concept of sensory integration therapy must be disseminated widely and correctly to meet these children's needs. Although most urban communities have a high number of children's spaces, these spaces require improvement. This study proposes the incorporation of the concept of sensory integration therapy into neighborhood open spaces for children to positively impact children's sensory development. The purpose of this study is to determine the effective facility factors of an occupational therapy room, translate them into a community facility design, clarify the categories and relative importance of each design attribute, and explore the design strategies of the children's facilities in neighborhood open spaces based on the sensory integration theory. This study investigates the importance of the sensory integration treatment level. The facilities in neighborhood open spaces for children can be considered systemic structures consisting of five partitioned units with different levels of importance among the synergistic components within each unit. These structures will enable children to experience sensory stimulation during daily outdoor play and will serve as preventive and therapeutic tools.

## 1. Introduction

Sensory integration refers to the integration of sensory information input by the brain from various parts of the human body that enables the body to respond to internal and external perceptions. Only through sensory integration, can different parts of the nervous system coordinate to produce a smooth interaction with the environment. A sensory integration disorder (SID) is diagnosed when the sensory system does not work properly [1]. According to Saeki et al. [2], SID can be divided into five categories: body movement coordination disorders, form and space perception disorders, body balance disorders, auditory-language disorders, and tactile defensiveness disorder. Sensory integration therapy provides sensory input from the vestibular system, muscles, joints, and skin, so that children can integrate these senses and generate adaptive responses [1]. Based on Ayres' theory, previous research has used appropriate motor responses combined with vestibular, proprioceptive, and tactile stimuli to improve children's academic performance, motor coordination, and language functions [3, 4]. SID occur mostly in children aged 3–12 years. The preventive stage of SID is before the age of three, the complementary stage of intervention is between the ages of three and six, and the optimal treatment stage is before the age of six [5].

At present, most sensory integration therapy is achieved through occupational therapy (OT), as proposed by Barton [6], and includes a rehabilitation process of applying purposeful and selected operational activities to evaluate, treat, and train patients who have lost the ability to take care of themselves and work in varying degrees due to physical, mental, and developmental dysfunctions or disabilities. The rate of SID among Chinese children aged 3–12 years ranges from 10% to 30% [7, 8], indicating a large demand for OT among Chinese children. Easy and frequent access to OT is an important factor in maintaining treatment outcomes and improving the well-being of patients with SID [9]. At present, hospitals in many areas of China have specialized OT rooms for sensory integration treatment for children; however, these hospitals are located in big cities and are very limited in number. For example, only four hospitals in Guangzhou have licensed OT rooms. In addition, the construction of an OT room costs more than 3 million yuan [10]. In addition, the concentration of medical resources makes it difficult for children to receive treatments conveniently and frequently, which often delays and interrupts treatments.

The main way for children to recognize the world is through play, and children's facilities in neighborhood open spaces are important to support play. In these facilities, children imitate reality with specific game behaviors, gain initial personal experiences, learn to recognize the environment, and develop additional abilities. Children's facilities in neighborhood open spaces can help parents and children establish a good parent-child relationship, provide parents with opportunities to communicate with their children, and help solve children's psychological problems in a healthy and effective way. Proper children's outdoor recreation facilities may prevent isolation, improve the psychological health of children, and meet children's cognition and willpower training [11]. Previous studies have focused on the planning and design of such facilities in neighborhood open spaces from the perspective of public health or human factors engineering [12]. However, children's facilities in most urban communities in China only meet the children's minimal need for outdoor activities due to efforts to control costs, and the number and types of children's facilities are insufficient in some communities where the design of the facilities is simple or in need of maintenance or updating. Furthermore, the area devoted to children's facilities is often insufficient and not interesting to children [13]. If the important features of sensory integration therapy facilities in OT rooms can be used in the design of children's facilities in neighborhood open spaces, these spaces will not only meet children's daily needs of outdoor activity but also provide preventive and therapeutic sensory activities. However, few studies have integrated the designs used in OT rooms based on sensory integration theories when planning children's facilities. It is necessary to determine what factors currently used in OT rooms are effective, identify the potential organizational structure relationships of these effective factors, determine what specific design features can be used in the design of children's facilities in neighborhood open spaces, and clarify which design features should be the core components of the design.

The purpose of this study is to determine the factors of OT rooms that are effective and interpret them into features that can be included in children's facilities in neighborhood open spaces. This study also clarifies the category and relative importance of each design feature attribute and explores the design strategy of children's facilities in neighborhood open spaces based on sensory integration theory. A hybrid multiple attribute decision-making (MADM) model was used. The fuzzy Delphi method (FDM) was used to extract the valid facility factors of OT rooms using a questionnaire based on expert experience, followed by the exploratory factor analysis (EFA) technique, applied to determine the validity and categories of each design feature. A design framework of the sensory integration therapy factors interpreted as design features of children's facilities in neighborhood open spaces was constructed. The DEMATEL-based ANP (DANP) technique was used to determine the most dominant influence points of design of children's facilities in neighborhood open spaces. Based on the results of the FDM-EFA-DANP analysis, the design strategies and basic guidelines for integrating OT into children's facilities in neighborhood open spaces were explored. The main contribution of this study is to combine sensory integration therapy with children's facilities in neighborhood open spaces, which provides a new perspective and specific design strategies for their design. The rest of the paper is organized as follows: Section 2 provides a review of previous researches, Section 3 makes a description of the research methods and steps applied in this study, Section 4 provides a graphical description of the results and discusses the design strategies, and Section 5 summarizes the contribution of the full text and clarifies the research limitations and future research.

## 2. Literature Review

### 2.1. The Application of the Sensory Integration Theory

American psychologist A. Jean Ayres first proposed the theory of sensory integration. At the beginning of the 20th century, studies regarding various fields of sensory integration were conducted in China. As theories of sensory integration emerged, the practices of related fields were enriched. An increasing number of scholars have recognized the necessity of sensory integration in children's daily lives and have undergone training related to therapies of the sensory integration theory. Sensory integration training has also been adopted by occupational therapists [14], providing an important knowledge and skill base for practitioners worldwide. The sensory integration theory is one of the earliest theories of operational therapy and guides clinicians when treating sensory disorders that affect performance while providing much evidence to validate its structure.

Ayres' early publications have contributed to the development of this theory since the 1950s, and it is one of the most cited and used theories in operational therapy [15]. In the 1970s, many Western countries established children's sensory integration training centers to providers regarding children's sensory integration abilities, especially those of preschool children. Studies have shown that over 90% of kindergartens in Western developed countries include children's sensory integration training [16]. In the 1980s, sensory integration training was incorporated into kindergartens in Asia. Japan and South Korea were the first countries to offer sensory integration training courses in kindergarten programs, followed by Hong Kong and Taiwan of China.

### 2.2. Related Research Regarding Sensory Integration

In recent years, there has been a growing interest in children's SID and sensory integration training leading to sensory integration rooms for children being included in medical institutions and kindergartens. However, some educators and parents of young children are underinformed regarding SID, and the number of therapists is limited, leading to numerous problems in the treatment of SID in some areas. Parents, schools, and medical institutions are not fully aware of the importance of sensory integration for children, especially in remote areas where medical conditions are not well developed. Due to the lack of professional guidance and of a unified, multidisciplinary approach, the number of treatment personnel is insufficient, and available treatments are incomplete. In addition, the price of sensory integration training rooms is too high for most areas, resulting in interrupted and ineffective treatments for children with fewer resources. Most research regarding sensory integration has been conducted in cities, with the inclusion of almost no rural children. Also, very few healthy children are included in studies regarding SID, as most studies only include patients with distinctive disorders. It is important to consider the requirements for sensory integration in healthy children when developing new facilities.

A review of previous research shows that sensory integration training is mainly conducted in sensory integration training rooms in rehabilitation schools, early education institutions, and hospitals. These settings are typically used for children with overt features of SID and families who take the initiative to treat their children with serious disorders. Sensory integration theory is being integrated into preschool education [17], and some parents and kindergarten teachers are conducting sensory integration therapy training [18].

Significant research has been conducted regarding the testing and refinement of the sensory integration assessment system [19], the influence of the sensory integration theory on sensory regulation and sleep habits in children with autism [20], the influence of the sensory integration theory on children with aphasia [21], the influence of the sensory integration theory on children with attention deficit hyperactivity disorder or learning disorders [22], and the relationship of the sensory integration theory with other medical fields such as neuromechanics [23]. The idea of sensory integration has been recognized in China for several years, though the development of sensory integration equipment has not been performed. Sensory integration activities have been included in an overall interactive game environment to enhance the effect of cultivating children's sensory integration abilities in Hong Kong, Macao, and Western countries, indicating that the research regarding sensory integration has made great progress, though many shortcomings remain.

### 2.3. Application of the Sensory Integration Theory in Related Fields

The sensory integration theory has been applied effectively in multiple related medical fields. The development of multisensory activities based on the theory of sensory integration has occurred for several years in some regions. These activities are mostly used in the fields of early childhood development and rehabilitation training of children, patients with reduced mobility, and elderly adults. Sensory integration activities enhance or soothe the sensory system using visual, auditory, tactile, and olfactory equipment. The new generation of sensory integration is based on the design of the sensory integration activity environment commonly used overseas and by the Hong Kong Occupational Therapists [24], whose concept differs from the sensory integration training environment used in China. In recent years, sensory integration activities have been applied to the training of children with dysthymia and in special physical education courses. The sensory integration theory can be helpful when designing children's educational products and be used to treat children with ADHD and other learning disorders. In addition, the theory of sensory integration can also be applied to the design of an interactive environment that can connect several sensory interactions into one activity, resulting in simultaneous multisensory and multisensational signals and feedback training.

## 3. Methods

Three quantitative analysis techniques from the field of operations research were used to form a hybrid MADM analysis model: the FDM, EFA, and DANP. The FDM was used to extract the effective facility factors that have an important influence in the OT room. This technology has been widely used in planning and evaluating research in related fields such as regional governance, community management, and landscape architecture [25, 26]. Compared with the traditional Delphi method, the advantages of the FDM include the reduction of the number of surveys, the complete expression of experts' opinions, rational experts that are solution-oriented, and an economical solution in terms of both time and cost. In this study, FDM technology was used to confirm the effectiveness of facility factors based on expert experience to establish a stable design framework [27]. EFA was used to determine the essential structure of multivariate observational variables and perform dimensionality reduction. In this study, the technology is used to classify the effective facility factors and summarize the relevant factors into the same dimension. Thirdly, in view of the correlation among the evaluation elements under the dimension, this study will apply DANP technology to clarify the influence network relationship and relative weight among the relevant elements. The application of this analysis technique relies on expert domain knowledge, through pairwise comparison between design criteria, to clarify the stakeholders' considerations of the relative importance of the criteria.

### 3.1. FDM Technique

**Figure 1:**
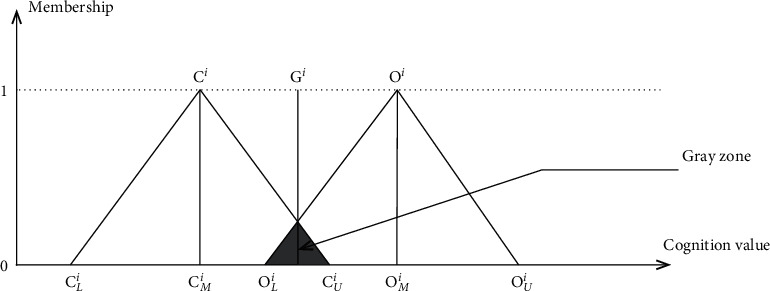
Triangular fuzzy numbers formed in the fuzzy Delphi method (FDM).

In this study, the FDM was used to integrate expert opinions using two triangular fuzzy numbers [28] and to determine whether expert cognition shows a consistent convergence effect using the gray zone verification method. The following three-step process was used:

Step (*F*1): The most conservative cognitive opinion and the most optimistic cognitive opinion provided by all experts to each factor *i* were statistically analyzed, and extreme values beyond two standard deviations were eliminated. Then, the minimum value **C**_*L*_^*i*^, geometric mean value **C**_*M*_^*i*^, and maximum value **C**_*U*_^*i*^ in the remaining most conservative cognitive value and the minimum value **O**_*L*_^*i*^, geometric mean value **O**_*M*_^*i*^, and maximum value **O**_*U*_^*i*^ in the most optimistic cognitive value were calculated.
Step (*F*2)*:* Based on the calculation results of step (*F*1), the three-angle fuzzy number **C**^*i*^=(**C**_*L*_^*i*^, **C**_*M*_^*i*^, **C**_*U*_^*i*^) of the most conservative cognition and the three-angle fuzzy number **O**^*i*^=(**O**_*L*_^*i*^, **O**_*M*_^*i*^, **O**_*U*_^*i*^) of the most optimistic cognition for each factor *i* were calculated (Figure 1).
Step (*F*3): Determining whether the experts' opinions present a consistent convergence effect can be accomplished in the following ways:
  (a) When no overlap between the two triangular fuzzy numbers is present (**C**_*U*_^*i*^ ≤ **O**_*L*_^*i*^), the opinion interval value of each expert includes a consensus section that the opinion tends to be within; the consensus value **G**_*U*_^*i*^ of this facility factor *i* is calculated usingfd1
    (1) $$\begin{array}{c} G_{U}^{i}=\frac{C_{M}^{i}+O_{M}^{i}}{2}. \end{array}$$
  (b) If there is an overlap between the two triangular fuzzy numbers (**C**_*U*_^*i*^ > **O**_*L*_^*i*^) and the gray area (**Z**^*i*^=**C**_*U*_^*i*^ − **O**_*L*_^*i*^) of the fuzzy relationship is smaller than the range **M**^*i*^=**O**_*M*_^*i*^ − **C**_*M*_^*i*^ between the geometric mean of optimistic cognition and the geometric mean of conservative cognition for the facility factor by the expert, there is no consensus section for each expert's opinion interval value; however, the two with extreme opinions (the most conservative opinion of the optimistic cognition and the most optimistic opinion of the conservative cognition) were not significantly different from other opinions, leading to divergent opinions. Therefore, the consensus value of this facility factor *i* is equal to the fuzzy set obtained by the intersection (minimum) operation of the fuzzy relation of two triangular fuzzy numbers. The quantization score of the fuzzy set with the maximum membership value can be obtained using equations (2) and (3).
    (2) $$\begin{array}{c} F^{i}\left(x_{j}\right)=\left\{\int_{x}\left\{\min\left[C^{i}\left(x_{j}\right),O^{i}\left(x_{j}\right)\right]\right\}\text{d}x\right\}, \end{array}$$
    (3) $$\begin{array}{c} G^{i}=\left\{x_{j}|\max\mu_{p^{i}}\left(x_{j}\right)\right\}. \end{array}$$
  (c) If there is an overlap between the two triangular fuzzy numbers, (**C**_*U*_^*i*^ > **O**_*L*_^*i*^), and the gray area (**Z**^*i*^=**C**_*U*_^*i*^ − **O**_*L*_^*i*^) of the fuzzy relationship is larger than the range **M**^*i*^=**O**_*M*_^*i*^ − **C**_*M*_^*i*^ between the geometric mean of optimistic cognition and the geometric mean of conservative cognition, there is no consensus section for each expert's opinion interval value, and the two extreme opinions differed too much from other opinions, leading to divergent opinions. Therefore, the questionnaires and FDM should be repeated until all the evaluation items reached convergence and a corresponding consensus value is obtained.

### 3.2. EFA Technique

The EFA is a dimension-reducing method of multivariate statistics that is used to identify the latent variables from manifest variables. A five-step process was used to complete the main procedure of the principal component analysis using EFA:

  Step (*E*1): The correlation matrix **R** or variance-covariance matrix for the objects to be assessed was found.
  Step (*E*2): The eigenvalues *λ*_*k*_,  *k*=1,2,…, *m* and eigenvectors *β*_*k*_=[*β*_1*k*_,…, *β*_*ik*_,…, *β*_*pk*_] were identified for the assessment of the factor loading $a_{ik}=\sqrt{\lambda_{k}}\beta_{ik}$ and the number of factors *m*.
  Step (*E*3): The eigenvalue ordering *λ*_1_ > ⋯>*λ*_*k*_ > ⋯>*λ*_*m*_, where *λ*_*m*_ > 1, was used to determine the number of common factors to be extracted by a predetermined criterion.
  Step (*E*4): According to Kaiser [29], the varimax element was used to find the rotated factor loading matrix, which provides additional insights for the rotation of the factor axis.
  Step (*E*5): The factor referring to the combination of manifest variables was named.

### 3.3. DANP Technique

The DANP technique was used to divide the relation matrix of the total influence from the first phase into influential weights (IWs, also termed global weights) of the criteria (vector *w*^*g*^) using the basic concept ANP to calculate the local weights of each criterion (vector *w*_*C*_^*l*^) and dimensions (vector *w*_*D*_^1^) [30]. Recently, this analysis technology has been widely used in the field of public health and urban planning. It can effectively integrate the experts' domain knowledge from stakeholders, clarify the impact network relationship and relative weight between evaluation criteria, and provide decision-makers with dynamic impact viewpoint [31–33].

  Step (*D*1): A direct influence relation matrix **A** was established based on questionnaire results. The assessment scores in the questionnaire included 0 (no influence), 1, 2, 3, and 4 (highly influential).
  Step (*D*2): The average direct influence relation matrix **E** was established. The average scores of the *H* experts were determined using *e*_*ij*_=(1/*H*)∑_*h*=1_^*H*^*a*_*i*,*j*_^*h*^.
  Step (*D*3): The consensus was examined using a threshold of the average gap ratio of 5%. Values less than 5% indicate a confidence level above 95%, which represents a stable system. When an unstable system is obtained, the first phase is implemented again to verify if the data were collected correctly and whether the number of experts was sufficient.
  Step (*D*4): The normalized average direct influence relation matrix **D** is formulated to remove the units and orientations of values and to convert the values of the numbers to between 0 and 1.
  Step (*D*5): The total influence relation matrix **T** was constructed using **T**=**D**(**I** − **D**)^−1^. This matrix served as the foundation for drawing the influential network relation map (INRM) in this study.
  Step (*D*6): The total influence relation matrix of criteria **T**_*C*_ and dimensions **T**_*D*_ were constructed.
  Step (*D*7): The unweighted supermatrix **W**^*a*^ was calculated.
  Step (*D*8): The weighted supermatrix **W** was calculated.
  Step (*D*9): The limit supermatrix was determined by multiplying the weighted supermatrix **W** by itself to achieve a convergent and stable matrix **W**^*g*^. The values in each column of the weighted supermatrix **W** represent the IWs.

## 4. Results and Discussion

### 4.1. Determining Which Factors in OT Rooms Are the Most Effective Using Expert Opinions

In this study, the patient room for surgery for four months was observed and recorded so as to collect the relevant information of patient-room facilities. Then, the qualitative data were obtained for this study from the collected photos, text memos, pictures, and videos in combination with relevant literature. The field investigation was carried out in the sensory integration treatment room of Guangzhou Rehabilitation Hospital. The relevant professional therapists were interviewed; we learnt about and experienced the common equipment and facilities in the training room. Finally, 27 facility factors were extracted from the treatment room (see supplementary file) based on the general inductive analysis method. Although continuous coding comparison has been carried out in the whole process of qualitative data induction and analysis, the effectiveness of these 27 convenience factors still needs to be tested by expert opinions. As described above, this study uses the FDM to integrate the domain knowledge of experts and selects the effective facility factors from the OT room on the basis of the consensus of experts.

The fuzzy Delphi questionnaire was completed by professional doctors and therapists who perform sensory integration therapy. All interviewees had at least five years of working experience, bachelor's degrees, and professional therapist qualification certificates. A total of 12 experts completed the questionnaire. After excluding invalid questionnaires, a total of 10 valid questionnaires were included in the analysis, including eight from female experts. One participating expert is a registered operative therapist, is master of operational therapy in Hong Kong, and has more than ten years of relevant practical and scientific research experience in this field. Based on the semantic discrimination of the five-point Likert scale, the threshold value of expert opinion was set at 6.667. When the validity of the facility factor was rated as less than 6.667, the facility factor was determined to be ineffective for the treatment of SID (Table 1).

### 4.2. Clarifying the Potential Hierarchical Relationship of Facility Factors in Sensory Integration Therapy

EFA was used to understand the content validity and potential hierarchical structure of the effective facility factors of the OT room, providing a basic reference for the construction of a framework for the design of children's facilities with sensory integration therapy in communities. From the perspective of community-based children's design, children's activity facilities should be viewed as a systematic whole, with well-organized subunits and synergistic interconnections among the components.

A questionnaire including items regarding the 20 facility factors was designed using a seven-point Likert-type scale and distributed to doctors and therapists involved in sensory integration therapy. Any unanswered questions were set as missing values. A total of 245 questionnaires were distributed, and 231 valid responses were received. The principal component analysis and the maximum variation method were used during data processing. Items with eigenvalues greater than 1 were selected, and those with a factor load less than 0.5 were rejected. The number of common factors was not limited. The 231 responses were found to be suitable for factor analysis (KMO = 0.980), with an internal consistency (Cronbach's *α*) of 0.977 among the 20 questionnaire items.

The 20 facility factors were categorized into five dimensions with a total cumulative explained variation of 78.723% (Table 2). As the factor loads of the square board swing and padded slipway were less than 0.5, these factors were excluded from the framework of sensory integration therapy for children. In this study, the following five dimensions were used: mobile experience series (**D**_1_), goal guidance series (**D**_2_), protection perception series (**D**_3_), limb coordination series (**D**_4_), and climbing series (**D**_5_). **D**_1_ includes five facility factors with an internal consistency of 0.918. **D**_2_ includes six facility factors with an internal consistency of 0.932. **D**_3_ includes three facility factors with an internal consistency of 0.864. **D**_4_ includes two facility factors with an internal consistency of 0.830. **D**_5_ includes two facility factors with an internal consistency of 0.827.

### 4.3. Clarifying the Influential Network Relation and IWs of the Design Criteria

After the clarification of the content validity and potential hierarchical structure of each facility factor, a focus group was formed including nine professional designers with similar educational backgrounds and experience in the design of open spaces in residential areas. A semistructured meeting was conducted regarding the 18 facility factors determined to be effective in sensory integration therapy, aiming to summarize and refine the design characteristics corresponding to each facility factor. This meeting resulted in a design framework for children's facilities that effectively integrates sensory integration therapy factors (Table 3). The design framework includes five facility dimensions, 18 facility factors, and corresponding facility design criteria. The construction of this hierarchical structure framework provides designers with a key design decision-making basis in practical engineering. The construction of this facility design framework enhances the designers' ability to control the overall morphological design of children's facilities in the community and guides the designers in the organization of the subunits of the children's facility system at the level of sensory integration therapy to stimulate creativity and enhance the synergy among the components of each unit.

**Figure 2:**
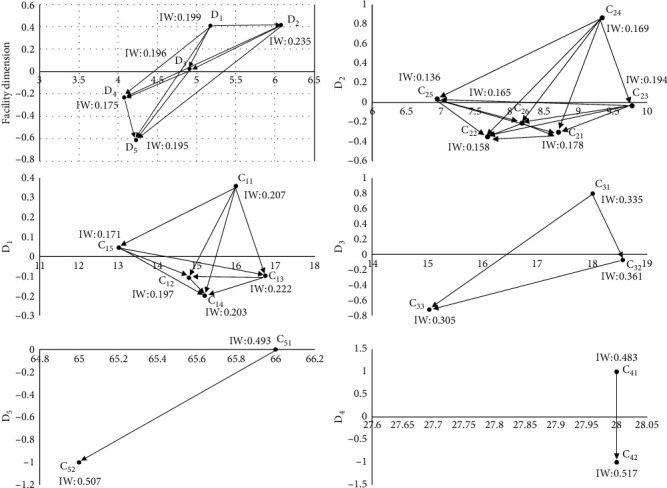
The influential network relation map (INRM) of total influence relationships.

In this study, DANP questionnaires were designed based on the construction of the design framework, which were used for the stakeholders from the circles of production, government, and academics. Most of the experts who received the questionnaires had participated in the design of children's activity facilities in the open space of urban neighborhood. Eventually, a total of 15 expert questionnaires were collected, of which 13 were valid and passed the consistency test by the expert opinions. The results of data analysis are shown in Figure 2. Among the five design dimensions, goal guidance (**D**_2_) has the highest influence weight, followed by **D**_1_, **D**_3_, **D**_5_, and **D**_4_ in turn. In this design framework system, **D**_2_ is the most influential design dimension. In **D**_2_ dimension, information-guided interactive game (**C**_23_) is the design criterion which has the most influence weight, but soft discussion with axle wheels for sliding (**C**_24_) is the most influential one. The influence weight and dominant influence of **D**_1_ are second only to **D**_2_. In the perspective of mobile experience (**D**_1_), children with dyskinesia were trained in physical motor coordination disorder, and the relative importance of **C**_1_ was the highest. **C**_1_ criterion refers to the children's activity facilities in the neighborhood open space, which can be used for children to grasp and slide, and suspended discussion with narrow and soft texture (**C**_11_) has the most dominant influence. **D**_5_ and **D**_4_ are the two design dimensions with the weakest influence weight and relatively weak dominant influence in this design framework.

In this study, the effective facility factors in the OT room were determined and the potential structural relationships of these facilities factors were explored. The main characteristics of facility design and the relative weight of design criteria were clarified, providing a key framework for the design of children's facilities that incorporate sensory integration therapy factors in neighborhood open spaces. Table 2 shows that the children's facilities in neighborhood open spaces are systemic structures that can be composed of five subunits. Each subunit serves a different purpose of sensory integration therapy, and the components of the facility within each unit remain synergistically related to each other. Based on the analysis results in Figure 2, this study integrates the influence weight and influence network relationship between design dimensions/criteria and explores the design strategy of children's facilities in open neighborhood space based on sensory integration theory. This design strategy can also be understood as a design guideline for children's facilities based on sensory integration theory or a decision-making proposal for launch of related design resources. In order to enhance the extensive sensory integration training, this study suggests that the design of children's facilities in neighborhood open space should focus on three aspects of goal guidance, mobile experience, and protection perception, so that the training facilities can be planned at different levels and the training contents of multiple mutual supporting can be output based on the law of sensory development of children with sensory disorders [34]. First of all, the most important design criterion in the goal guidance unit is to guide children to make corresponding behavior activities in the interactive game by receiving the output of information through hearing and identifying it through brain. In the process of voice perception, children's ability to integrate auditory and visual information is trained to improve their ability of voice understanding. It is very important in the early language development of children with SID [35]. In addition, this study suggests that designers should also consider children's sitting, lying, sliding, and hanging facilities as the basis of the system based on the design of children's mobile experience in neighborhood open space. With the help of effective guidance, children can make many corresponding behavioral activities including sliding, climbing, and rotation.

Secondly, designers should attach importance to the application of virtual reality technology and the influence of video games in the facility system. Virtual reality technology can be regarded as an ideal tool for children to practice their behavior in the situation of role playing, and it also provides safe environment for rule learning and task repetition [36]. Dana et al. [37] have found that virtual reality game intervention lasting for 12 weeks could effectively improve the dynamic balance of children with developmental coordination disorder. In addition, in terms of protection perception facilities, designers should provide luminous game props of different colors and shapes and focus on the design of cushion facilities to protect children's safety when they are on the ground or hanging in the air. The environment of training space should cater to the SID children with various habits and movement tendencies. For the sake of safety, walls and floors need to be covered with soft materials, and the surface in the space is composed of soft objects, such as large cushions and flexible units of cushions of various shapes [38]. Children's activity space and all its elements should provide a variety of sensory inputs (vision, touch, and hearing) to provide children with the opportunities to explore their body consciousness and balance as much as possible. On the whole, designers should try to make children understand the narration of movement space, involving the single movement of children and the operation of triggering subsequent movement in the movement. In terms of body coordination and climbing training, designers need to make the supporting surface for climbing and the lower surface for landing clear and focus on supporting the body in different directions to promote children's sense of balance and functional training of vestibular system.

## 5. Conclusion

In this study, the effective facility factors of an OT room were identified, categorized, and incorporated into a design framework for children's facilities that include sensory integration therapy in neighborhood open spaces. Through a general inductive analysis, 27 facility factors were extracted and the FDM was used to select 20 factors of OT rooms that were thought to be effective by experts. To further confirm the content validity and potential structural relationship of these effective facility factors, EFA was used to determine five categories of facility factors, and 18 facility factors were extracted. Finally, the design characteristics of each facility factor were summarized to construct a framework of children's facility design with sensory integration therapy. Finally, in view of the correlation among the evaluation elements under the dimension, this study will apply DANP technology to clarify the influence network relationship and relative weight among the relevant elements.

The analysis results of the above research stages are the suggestions in the segmentation and supplement of the design criteria on the basis of the relevant current provisions of game facilities in *Code for Planning and Design of Urban Residential Areas* and *Code for Design of Parks* in China. A total of 5 facility dimensions and 18 facility factors will be taken as important design criteria and will be used by designers in the design strategy of existing children's facility transformation and new product development in a priority way based on the weight and actual implementation conditions. Based on such modes as literature review, questionnaire survey, expert interview, and field research, this study proves that it is feasible to apply indoor sensory integration training equipment and facilities to outdoor children's space, and it not only reduces the burden of medical staff and accompanying family members to a large extent but also helps children to receive sensory stimulation and improve their skills in daily life activities. The purpose of the study is to further solve the difficulties of children in the process of sensory integration therapy. Additionally, it is the first attempt to combine theory with practical medical device design in children's space facilities in community in China, which is undoubtedly the convenience for the majority of children. In the future study, the real-time feedback and evaluation will be followed up, and it should be noted that the design criteria of facilities with the same dimensions should be more closely combined or placed together to facilitate the use of the facilities and the enhancement of effectiveness. Furthermore, it should be noted that the influence of outdoor environmental factors should be considered and that how to use more weather resistant facility materials and form relationship while ensuring that the design criteria will not be affected by the outdoor environment factors will become one of the key points of further research in this paper. In addition, based on the results of this study, a number of different design schemes can be made in the future according to the specific design strategies of children's facilities in the display situation. The performance of the design scheme before construction can be analyzed from the perspective of sensory integration therapy by using the appropriate evaluation model.

## Figures and Tables

**Table 1:** Validity screening of sensory integration therapy facility factors.

Facility factor	Conservative value **C**^*i*^		Optimistic value **O**^*i*^		Single value **a**^*i*^		Geometric mean **M**		Calibration value		Consensus value
	Min	Max	Min	Max	Min	Max	**C** ^*i*^	**O** ^*i*^	**a** ^*i*^	**M**_*i*_–**Z**_*i*_	**G** ^*i*^
Soft building block house	3	7	6	10	4	9	5.161	7.191	6.295	1.030	**6.393**
Hot dog cushion	3	8	5	9	5	8	4.752	7.846	6.406	0.094	**6.401**
Sponge mat	3	9	7	9	4	9	5.568	8.395	6.227	0.827	7.578
Soft building blocks	2	10	7	10	6	10	5.725	9.068	7.306	0.342	7.978
Patterned soft cubes	1	7	5	7	2	7	3.322	6.450	4.648	1.128	**5.566**
Beanbag chair	1	8	6	8	4	8	3.651	6.959	6.007	1.308	**6.361**
Rotary drum	2	8	6	8	5	8	5.453	7.533	6.586	0.080	6.751
Folding ball pool	3	8	5	10	4	8	4.637	7.705	5.685	0.069	**6.337**
Wooden pull-out bed magic board	3	8	7	10	5	9	5.709	8.347	7.163	1.638	7.370
Back magic board wooden ladder	2	8	6	10	4	9	5.646	7.804	7.186	0.159	6.868
Back magic board rope net	3	8	7	10	5	8	5.473	8.243	7.075	1.769	7.330
Climbing wall	3	8	6	9	5	8	5.119	8.075	6.488	0.956	6.838
Balancing swing	2	8	6	10	5	8	5.241	7.566	6.342	0.325	6.724
Pumpkin swing	3	8	5	10	5	9	4.643	7.714	6.624	0.071	**6.341**
Square board swing	3	9	7	10	4	10	5.735	8.763	7.447	1.028	7.701
Sliding swing	3	9	7	9	4	10	6.209	8.415	7.468	0.206	7.673
Spinning top	3	8	6	10	4	9	5.301	8.198	6.533	0.897	6.898
Hanger rope	3	9	7	10	4	10	6.389	8.423	7.359	0.034	7.706
Padded-side bouncing bed	3	8	6	9	5	9	5.404	7.708	6.853	0.305	6.794
Padded slipway	3	8	6	9	5	8	4.743	7.708	6.722	0.966	6.688
Skateboard	4	9	7	9	5	9	6.106	8.463	6.822	0.357	7.672
Ball chair	4	8	7	10	7	9	5.876	8.347	7.683	1.471	7.388
Challenging facilities	5	9	7	9	6	10	6.418	8.469	7.390	0.050	7.725
Fruit-featured beanbag	4	8	7	10	6	10	6.439	8.241	7.590	0.802	7.443
Light-sensing game board	4	9	7	10	6	9	5.640	8.204	7.499	0.564	7.527
Spinning mirror ball	5	7	6	9	6	9	6.084	7.516	7.661	0.431	**6.623**
Virtual game	4	7	7	9	6	8	5.596	8.139	6.971	**C**_*U*_^*i*^ ≤ **O**_*L*_^*i*^	6.868
Number of valid facility factors selected				**21**			Consensus threshold value				**6.667**

**Table 2:** Factor analysis of facility factors in sensory integration therapy.

(*N* = 231) Facility factors	Facility dimension				
	Mobile experience (**D**_1_)	Goal guidance (**D**_2_)	Protection perception (**D**_3_)	Limb coordination (**D**_4_)	Climbing (**D**_5_)
Balancing swing	0.698				
Soft building blocks	0.624				
Sliding swing	0.582				
Ball chair	0.581				
Virtual game	0.542				
Rotary drum		0.690			
Spinning top		0.617			
Challenging facility		0.594			
Skateboard		0.592			
Wooden pull-out bed magic board		0.567			
Climbing wall		0.541			
Sponge mat			0.687		
Hanger rope			0.676		
Light-sensing game board			0.507		
Square board swing					
Fruit-featured beanbag				0.703	
Padded-side bounce bed				0.548	
Padded slipway					
Back magic board wooden ladder					0.668
Back magic board rope net					0.541
**Cronbach *α***	**0.918**	**0.932**	**0.864**	**0.830**	**0.827**
**Cumulative Variance**	**78.723%**				

**Table 3:** Design framework of children's facilities based on sensory integration theory.

Facility dimension	Facility factor	Design criteria
Mobile experience (**D**_1_)	Balancing swing	Suspended cushion with narrow and soft texture (**C**_11_)
	Soft building blocks	Soft building blocks with different color and shape (**C**_12_)
	Sliding swing	For children to sit on and slide when grasping a rope (**C**_13_)
	Ball chair	Gliding seat with axle wheel (**C**_14_)
	Virtual game	Video game with virtual scene (**C**_15_)

Goal guidance (**D**_2_)	Rotary drum	Hollow cylinder for children's multifaceted physical interaction (**C**_21_)
	Spinning top	Hard object for children to sit on or lie in and rotate (**C**_22_)
	Challenging facility	Information-guided interactive game (**C**_23_)
	Skateboard	Soft cushion with axle wheels for sliding (**C**_24_)
	Wooden pull-out bed magic board	Suspended cradle for children to lie down (**C**_25_)
	Climbing wall	Wall to guide the children for climbing (**C**_26_)

Protection perception (**D**_3_)	Sponge mat	Soft cushion to give children protection on the ground (**C**_31_)
	Hanger rope	Suspended hammock that can be moderately swung (**C**_32_)
	Light-sensing game board	Different colors and shapes of visual light-emitting components (**C**_33_)

Limb coordination (**D**_4_)	Fruit-featured beanbag	Graspable fruit-featured small soft object (**C**_41_)
	Padded-side bounce bed	Elastic net with soft padded enclosure for children to bounce vertically (**C**_42_)

Climbing (**D**_5_)	Back magic board wooden ladder	Ladder-like structure designed to fix the climbing rhythm and direction (**C**_51_)
	Back magic board rope net	Rope net structure designed with picking targets (**C**_52_)

## Data Availability

The data used to support the findings of this study are included in the article.

## Supplementary Materials

Supplementary Materials This is an introduction to the 27 facility factors mentioned in the paper: (1) soft building blockhouse (available to stimulate the body sense organ of the child and actively control the message accepted by the body sense organ), (2) hot dog cushion (the child lies down in the middle of the soft mat and experiences the feeling of being wrapped up with stimulation provided to its vestibule), (3) sponge mat (offer sufficient protection to the child during the training period), (4) soft building blocks (the game player combines the props in different forms based on the color and shapes; it is available for multiple players to play the game together, thereby guiding the players in the training activities for large muscle and strengthening the ability of the sense of balance), (5) patterned soft cubes (used for early childhood education; by breaking the traditional teaching theory for woodblocks, the soft blocks can protect the child during activity; moreover, the number on the blocks are available for the training of cognitive abilities of the child), (6) beanbag chair (the game player sits on the bean bag chair, thus stimulating the tactile sensory system of the player), (7) rotary drum (it is a balance game in which the player can grovel or stand on the roller, thereby promoting the overall development of the vestibular function and improving the inherent feeling and tactile stimulus of the vestibule in the meantime), (8) folding ball pool (it consists of heaped plastic small balls in different colors with moderate hardness, which is available to improve tactile sensitiveness or insufficiency: strengthening vestibular balance, physical coordination ability, and brain cadres function), (9) wooden pull-out bed magic board (experience the feeling of lying in the cradle in the infantile period, thus improving vestibular sensation and releasing muscle overstrain), (10) back magic board wooden ladder (available to provide diversified and challenging kinesthesia training for the child with sensory integration dysfunction of different level), (11) back magic board rope net (available to provide diversified and challenging kinesthesia training for the child with sensory integration dysfunction of different level), (12) climbing wall (it is a guided target remotely controlled by the tutor, which is available to provide diversified and challenging kinesthesia training for the child with sensory integration dysfunction of different level), (13) balancing swing (let the child sit on the lathy swing to strengthen the coordinating and balancing ability of the body), (14) pumpkin swing (improve the activity of the inherent sensory system of the vestibule and strengthen the tactile system; improve the body coordination of the child through acquiring a large amount of vestibule information, which is helpful for the inherent sensation input integration of the vestibule; it is also beneficial for the development of limb strength of the child), (15) square board swing (it is a coaxial rotary swing, on which the player can pose differently, including lying on its back or stomach, sitting or standing up, and so on; the tactile mat is available to provide more comfort tactile relaxation feeling to some players who suffer from tactile sensory disorder), (16) sliding swing (it works together with the dual-layer game platform and the ball pool and forms an interesting sensation integration game, thereby facilitating the learning of vestibular sensation, vision, proprioception, and tactile sensation), (17) spinning top (in sensation integration training, body large gyroscope, rotary cylinder, carousel-carrousel, rotary chair, and so on are all used to train vestibular balance), (18) hanger rope (it promotes the normalization of the vestibular system of the child by inputting appropriate sensation of rocking to the child), (19) padded-side bouncing bed (the child observes the surrounding environment and implements goal-response action when bouncing horizontally), (20) padded slipway (the child glides downward lying on its stomach with the skidding board placed on the skidding platform), (21) skateboard (the child glides lying on its stomach on the skidding board, thereby stimulating the vestibular system and the proprioceptive sense and promoting the double-side coordination of the body), (22) ball chair (it helps the child to build its vestibular sensory function, control the the sense of gravity, and develop the balance ability of the child; it has the best effect on the child with hyperactivity or poor coordination ability; in the meantime, it is also beneficial for the exercising of the child's waist and abdomen strength), (23) challenging facilities (improve the body coordination of the child through acquiring a large amount of vestibule information, which is helpful for the inherent sensation input integration of the vestibule; it is also beneficial for the development of limb strength of the child), (24) fruit-featured beanbag (improve the visual discrimination ability, fine operation ability, and reactivity through instructions), (25) light-sensing game board (train the abilities for cognition, language understanding, expression, and eye-hand coordination through visual sensation feelings of light), (26) spinning mirror ball (the light and shadow reflected by the rotating of the mirror ball are available to motivate the player with a weak sense of sight and help them to increase the attention to the surrounding environment, thereby improving the ability for spatial judgment and communication), and (27) virtual game (implement 3D interaction with different gaming methods and restore the real situation in a combination of AR technology augmented reality; make improvements in different aspects, including social communication, emotion, and feeling).
