# Supplementary material for: Application of Hybrid Multiple Attribute Decision-Making Model to Explore the Design Strategies of Children's Facilities in Neighborhood Open Spaces Based on Sensory Integration Theory
Source: J Healthc Eng. 2021 May 29;2021:5556172. doi: 10.1155/2021/5556172 (PMC8181119; doi:10.1155/2021/5556172)
Supplement: Supplementary Materials — This is an introduction to the 27 facility factors mentioned in the paper: (1) soft building blockhouse (available to stimulate the body sense organ of the child and actively control the message accepted by the body sense organ), (2) hot dog cushion (the child lies down in the middle of the soft mat and experiences the feeling of being wrapped up with stimulation provided to its vestibule), (3) sponge mat (offer sufficient protection to the child during the training period), (4) soft building blocks (the game player combines the props in different forms based on the color and shapes; it is available for multiple players to play the game together, thereby guiding the players in the training activities for large muscle and strengthening the ability of the sense of balance), (5) patterned soft cubes (used for early childhood education; by breaking the traditional teaching theory for woodblocks, the soft blocks can protect the child during activity; moreover, the number on the blocks are available for the training of cognitive abilities of the child), (6) beanbag chair (the game player sits on the bean bag chair, thus stimulating the tactile sensory system of the player), (7) rotary drum (it is a balance game in which the player can grovel or stand on the roller, thereby promoting the overall development of the vestibular function and improving the inherent feeling and tactile stimulus of the vestibule in the meantime), (8) folding ball pool (it consists of heaped plastic small balls in different colors with moderate hardness, which is available to improve tactile sensitiveness or insufficiency: strengthening vestibular balance, physical coordination ability, and brain cadres function), (9) wooden pull-out bed magic board (experience the feeling of lying in the cradle in the infantile period, thus improving vestibular sensation and releasing muscle overstrain), (10) back magic board wooden ladder (available to provide diversified and challenging kinesthesia training for [file 5556172.f1.doc]

| **Facility image** | **Facility description** | **Facility image** | **Facility description** |
| --- | --- | --- | --- |
| 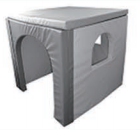 | **1.** **Soft building block house**  (length: 900mm; width:900mm; height:900mm)  Available to stimulate the body sense organ of the child, and actively control the message accepted by the body sense organ. | 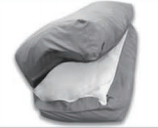 | **2. Hot dog cushion**  The child lies down in the middle of the soft mat, and experiences the feeling of being wrapped up with stimulation provided to its vestibule. |
| 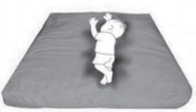 | **3. Sponge mat**  (length: 1500mm; width: 1500mm; height: 200mm)  Offer sufficient protection to the child during the training period. | 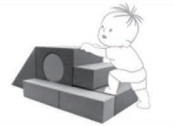 | **4.** **Soft building blocks**  (length: 1700mm; width: 1000mm; height: 600mm)  The game player combines the props in different forms based on the color and shapes. It is available for multiple players to play the game together, thereby guiding the players in the training activities for large muscle, and strengthening the ability of the sense of balance. |
| 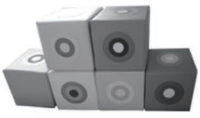 | **5. Patterned soft cubes**  (length: 300mm; width:300mm; height:300mm)  Used for early childhood education. By breaking the traditional teaching theory for wood blocks, the soft blocks can protect the child during its activity. Moreover, the number on the blocks are available for the training of cognitive abilities of the child. | 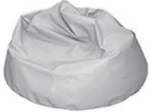 | **6. Beanbag chair**  (upper diameter: 250mm; lower diameter: 1000mm; height:900mm)  The game player sits on the bean bag chair, thus stimulating the tactile sensory system of the player. |
| 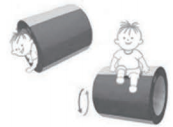 | **7. Rotary drum**  (outer diameter: 700mm; inner diameter: 500mm; length: 900mm)  It is a balance game in which the player can grovel or stand on the roller, thereby promoting the overall development of the vestibular function, and improving the inherent feeling and tactile stimulus of the vestibule in the meantime. | 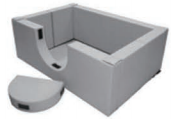 | **8. Folding ball pool**  (length: 1500mm; width: 1000mm; height: 500mm)  It consists of heaped plastic small balls in different colors with moderate hardness, which is available to improve tactile sensitiveness or insufficiency: strengthening vestibular balance, physical coordination ability and brain cadres function. |
| 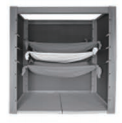 | **9.** **Wooden pull-out bed magic board**  (length: 2400m; width: 200mm; height: 2400mm)  Experience the feeling of lying in the cradle in the infantile period, thus improving vestibular sensation, and releasing muscle overstrain. | 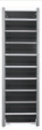 | **10.** **Back magic board wooden ladder**  (length: 700mm; height: 2200mm)  Available to provide diversified and challenging kinesthesia training for the child with sensory integration dysfunction of different level. |
| 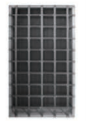 | **11.** **Back magic board rope net**  (length: 1200mm; height: 2200mm)  Available to provide diversified and challenging kinesthesia training for the child with sensory integration dysfunction of different level. | 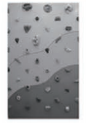 | **12.** **Climbing wall**  (length: 3000mm; height: 2400mm)  It is a guided target remotely controlled by the tutor, which is available to provide diversified and challenging kinesthesia training for the child with sensory integration dysfunction of different level. |
| 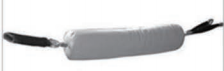 | **13. Balancing swing**  (diameter: 250mm; length: 1200mm)  Let the child sit on the lathy swing to strengthen the coordinating and balancing ability of the body. | 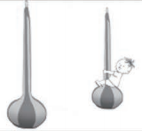 | **14. Pumpkin swing**  (diameter: 500mm; height: 1500mm)  Improve the activity of the inherent sensory system of the vestibule, and strengthen the tactile system. Improve the body coordination of the child through acquiring large amount of vestibule information, which is helpful for the inherent sensation input integration of the vestibule. It is also beneficial for the development of limb strength of the child. |
| 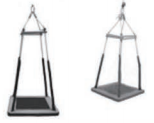 | **15. Square board swing**  (length: 700mm; width: 700mm; height: 1500mm)  It is a co-axial rotary swing, on which the player can pose differently, including lying on its back or stomach, sitting or standing up, etc. The tactile mat is available to provide more comfort tactile relaxation feeling to some players who suffer from tactile sensory disorder. | 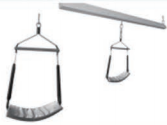 | **16. Sliding swing**  (length: 670mm; width: 250mm; height: 1200mm)  It works together with the dual-layer game platform and the ball pool, and form an interesting sensation integration game, thereby facilitating the learning of vestibular sensation, vision, proprioception and tactile sensation. |
| 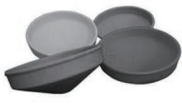 | **17. Spinning top**  (diameter: 800mm; height: 400mm)  In sensation integration training, body large gyroscope, rotary cylinder, carousel-carrousel, rotary chair, etc. are all used to train vestibular balance. | 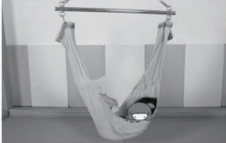 | **18. Hanger rope**  (the height is deemed to be appropriate when the lowest point is 30~40cm to the ground at the time of rocking) It promotes the normalization of the vestibular system of the child by inputting appropriate sensation of rocking to the child. |
| 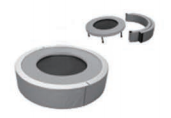 | **19.** **Padded-side bouncing bed**  (diameter: 1150mm; height: 50mm)  The child observes the surrounding environment, and implement goal-response action when bouncing horizontally. | 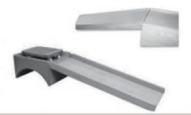 | **20. Padded slipway**  (length: 2180mm; width: 605mm; height: 425mm)  The child glides downward lying on its stomach with the skidding board placed on the skidding platform. |
| 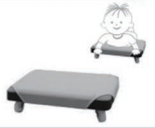 | **21. Skateboard**  (length: 560mm; width: 380mm; height: 150mm)  The child glides lying on its stomach on the skidding board, thereby stimulating the vestibular system and the proprioceptive sense, and promoting the double-side coordination of the body. | 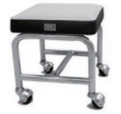 | **22. Ball chair**  (length: 310mm; width: 310mm; height: 350mm)  It helps the child to build its vestibular sensory function, control the the sense of gravity, and develop the balance ability of the child. It has the best effect on the child with hyperactivity or poor coordination ability, in the meantime, it is also beneficial for the exercising of the child’s waist and abdomen strength. |
| 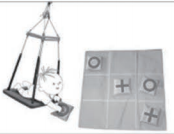 | **23. Challenging facilities**  (length: 900mm; width: 900mm)  Improve the body coordination of the child through acquiring large amount of vestibule information, which is helpful for the inherent sensation input integration of the vestibule. It is also beneficial for the development of limb strength of the child. | 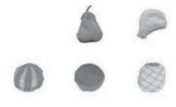 | **24.** **Fruit-featured beanbag**  (the fruits are in different shapes, approximately 110mm*120mm)  Improve the visual discrimination ability, fine operation ability and reactivity through instructions. |
| 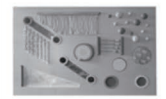 | **25. Light-sensing game board**  (length: 1500mm; height: 1000mm; thickness: 150mm)  Train the abilities for cognition, language understanding, expression and eye-hand coordination through visual sensation feelings of light. | 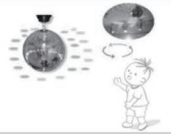 | **26. Spinning mirrorball**  (diameter: 300mm)  The light and shadow reflected by the rotating of the mirror ball are available to motivate the player with weak sense of sight, help them to increase the attention to surrounding environment, thereby improving the ability for spatial judgment and communication. |
| 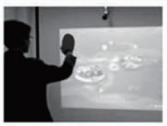 | **27.** **Virtual game**  Implement 3D interaction with different gaming methods, and restore the real situation in combination of AR technology augmented reality. Make improvements in different aspects, including social communication, emotion, feeling, etc. |  |  |
